# Fluoroquinolones and Biofilm: A Narrative Review

**DOI:** 10.3390/ph17121673

**Published:** 2024-12-11

**Authors:** Nicholas Geremia, Federico Giovagnorio, Agnese Colpani, Andrea De Vito, Alexandru Botan, Giacomo Stroffolini, Dan-Alexandru Toc, Verena Zerbato, Luigi Principe, Giordano Madeddu, Roberto Luzzati, Saverio Giuseppe Parisi, Stefano Di Bella

**Affiliations:** 1 Unit of Infectious Diseases, Department of Clinical Medicine, Ospedale “dell’Angelo”, 30174 Venice, Italy; 2 Unit of Infectious Diseases, Department of Clinical Medicine, Ospedale Civile “S.S. Giovanni e Paolo”, 30122 Venice, Italy; 3 Department of Molecular Medicine, University of Padua, 35121 Padua, Italy; federico.giovagnorio@studenti.unipd.it (F.G.); saverio.parisi@unipd.it (S.G.P.); 4 Unit of Infectious Diseases, Department of Medicine, Surgery and Pharmacy, University of Sassari, 07100 Sassari, Italy; colpaniagnese@gmail.com (A.C.); andreadevitoaho@gmail.com (A.D.V.); giordano@uniss.it (G.M.); 5 Faculty of Medicine, Iuliu Hatieganu University of Medicine and Pharmacy, 400012 Cluj-Napoca, Romania; botan.alexandru@elearn.umfcluj.ro; 6 Department of Infectious-Tropical Diseases and Microbiology, IRCCS Sacro Cuore Don Calabria Hospital, Negrar, 37024 Verona, Italy; giacomo.stroffolini@gmail.com; 7 Department of Microbiology, Iuliu Hatieganu University of Medicine and Pharmacy, 400012 Cluj-Napoca, Romania; toc.dan.alexandru@elearn.umfcluj.ro; 8 Infectious Diseases Unit, Trieste University Hospital (ASUGI), 34125 Trieste, Italy; verena.zerbato@gmail.com; 9 Clinical Microbiology and Virology Unit, Great Metropolitan Hospital “Bianchi-Melacrino-Morelli”, 89128 Reggio di Calabria, Italy; luigi.principe@ospedalerc.it; 10 Clinical Department of Medical, Surgical and Health Sciences, Trieste University, 34129 Trieste, Italy; roberto.luzzati@asugi.sanita.fvg.it (R.L.); stefano932@gmail.com (S.D.B.)

**Keywords:** fluoroquinolone, biofilm, biofilm-forming infections, fluoroquinolone and biofilm, antibiotic therapy

## Abstract

**Background**: Biofilm-associated infections frequently span multiple body sites and represent a significant clinical challenge, often requiring a multidisciplinary approach involving surgery and antimicrobial therapy. These infections are commonly healthcare-associated and frequently related to internal or external medical devices. The formation of biofilms complicates treatment, as they create environments that are difficult for most antimicrobial agents to penetrate. Fluoroquinolones play a critical role in the eradication of biofilm-related infections. Numerous studies have investigated the synergistic potential of combining fluoroquinolones with other chemical agents to augment their efficacy while minimizing potential toxicity. Comparative research suggests that the antibiofilm activity of fluoroquinolones is superior to that of beta-lactams and glycopeptides. However, their activity remains less effective than that of minocycline and fosfomycin. Noteworthy combinations include fluoroquinolones with fosfomycin and aminoglycosides for enhanced activity against Gram-negative organisms and fluoroquinolones with minocycline and rifampin for more effective treatment of Gram-positive infections. Despite the limitations of fluoroquinolones due to the intrinsic characteristics of this antibiotic, they remain fundamental in this setting thanks to their bioavailability and synergisms with other drugs. **Methods**: A comprehensive literature search was conducted using online databases (PubMed/MEDLINE/Google Scholar) and books written by experts in microbiology and infectious diseases to identify relevant studies on fluoroquinolones and biofilm. **Results**: This review critically assesses the role of fluoroquinolones in managing biofilm-associated infections in various clinical settings while also exploring the potential benefits of combination therapy with these antibiotics. **Conclusions**: The literature predominantly consists of in vitro studies, with limited in vivo investigations. Although real world data are scarce, they are in accordance with fluoroquinolones’ effectiveness in managing early biofilm-associated infections. Also, future perspectives of newer treatment options to be placed alongside fluoroquinolones are discussed. This review underscores the role of fluoroquinolones in the setting of biofilm-associated infections, providing a comprehensive guide for physicians regarding the best use of this class of antibiotics while highlighting the existing critical issues.

## 1. Introduction

Quinolones are a family of antibiotics containing a bicyclic core structure related to the compound 4-quinolone. The first quinolone, nalidixic acid, was discovered in 1962 [1]. In the 1970s–1980s, the quinolone class expanded significantly with the development of fluoroquinolones (FQs), which demonstrate a much broader spectrum of activity and improved pharmacokinetics and pharmacodynamics (PK/PD) compared to the first-generation quinolones [1,2]. For over five decades, FQs have been favored as antibiotics due of their high potency, broad-spectrum activity, favorable bioavailability, convenient formulations, high serum concentrations, high tissue penetrations and comparatively low incidence of side effects [1,3].

The term “biofilm”, originally used in technical and environmental microbiology, was introduced into medicine in 1982 following observations that *Staphylococcus aureus* developed a biofilm on a cardiac pacemaker [4]. Many bacteria can switch from the solitary planktonic bacterial lifestyle to the communal biofilm lifestyle. Biofilm eradication presents a significant challenge for clinicians. Some antibiotics require active cell growth to exert bactericidal effects. For example, penicillin is ineffective against nongrowing cells, while FQs can kill nongrowing bacteria but are more effective against rapidly dividing cells. Consequently, slow growth contributes to antibiotic resistance [5].

FQs may be a valuable option in many biofilm-forming infections due to their in vitro activity against biofilm formation. In vitro and in vivo data showed good biofilm eradication rates when FQs were compared to other antibiotics, mainly for Pseudomonas aeruginosa, Escherichia coli and Stenotrophomonas maltophilia [6]. FQs exert bactericidal killing activity against nongrowing bacteria and show a distinct efficacy against sessile cells in mature biofilms due to their excellent penetration rate into exopolysaccharides (EPSs) [5].

Although FQs are an excellent weapon against biofilm-forming diseases, in recent years, the Food and Drug Administration (FDA) has published drug safety communication about safety information regarding the risk of severe side effects associated with FQs administration. FDA-boxed warnings about FQs include the increasing risk of hypoglycemic coma, mental health side effects, increased risk of musculoskeletal diseases, such as tendinitis and tendon rupture, worsening symptoms for myasthenia gravis, the potential for irreversible peripheral neuropathy and the risk of aortic aneurysm rupture [7]. The high risk of side effects has limited the use of FQs in recent years, reserving this class of antibiotics only in case of severe infections or conditions with no alternative treatment options [7]. Otherwise, FQs have intrinsic fragility and antibiotic resistance induction that could be a serious problem. In some cases, a single-point mutation could be sufficient to cause a resistance profile in this class of antibiotics, reducing susceptibility to FQs [8].

Despite these concerns, FQs are still commonly used in real world clinical practice, especially in infections that benefit from lipophilic drugs (e.g., osteomyelitis) or as a step down from intravenous to oral therapy. They remain a valuable option for biofilm-associated infections, particularly caused by *Pseudomonas* spp.

Previous reviews have summarized the basic properties of FQs (e.g., pharmacokinetics and spectrum of activity) [9], their environmental impact and role in fostering antimicrobial resistance through ecological accumulation [10] and structural advancements within the class [11], providing a broader context for understanding the relevance and challenges associated with these agents. This review aims to provide the latest evidence on the role of FQs in biofilm-associated infections, with a particular focus on their comparative efficacy, combination therapies and challenges in clinical applications.

## 2. Pharmacology and Characteristics of Fluoroquinolones

**Figure 1:**
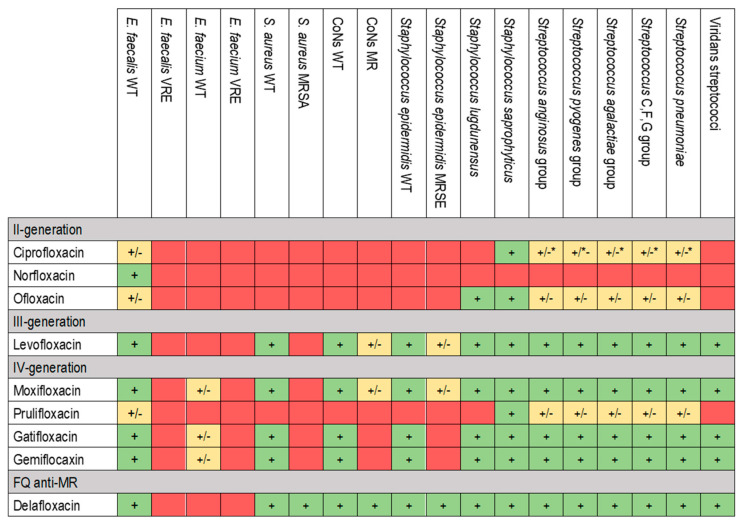
Aerobic Gram-positive fluoroquinolones spectrum. In green, the agent is active in vitro, and clinical studies have confirmed its activity against the microorganism; in yellow, the agent has limited or variable activity against the bacteria; in red, the agent is not active. +: susceptible, +/-: limited utility, CoNs: coagulase negative staphylococci, FQ: fluoroquinolone, MR: methicillin-resistant, MRSA: methicillin-resistant *Staphylococcus aureus*, MRSE: methicillin-resistant *Staphylococcus epidermidis*, VRE: vancomycin-resistant *Enterococcus* and WT: wild-type. * Controversial use, better not use.

FQs are one of the most prescribed antimicrobial agents. They represent the third largest group of antibacterial drugs, thus constituting 17% of the worldwide market sales [12]. FQs have a bactericidal effect on Gram-positive and Gram-negative bacteria, because their mechanism of activity relies on targeting two enzymes essential for DNA replication and repair: DNA gyrase and DNA topoisomerase IV [13,14,15]. DNA gyrase and topoisomerase IV form cleavage complexes by forming covalent bonds between active site tyrosine residues and 5′ overhangs at the DNA break. FQs hinder this crucial process by blocking DNA strand rejoining through reversible noncovalent binding with the cleavage complex at the cleavage–ligation active site [16]. This interaction leads to the formation of the quinolone–topoisomerase–DNA ternary complex, halting the DNA replication machinery [17]. The breaks in the DNA strands trigger the cascade of proteins under the SOS response, which, together with the extension of inadequate DNA repair, ultimately leads to the bactericidal activity of FQs [18,19]. Figure 1 reports the aerobic Gram-positive FQs spectrum.

**Figure 2:**
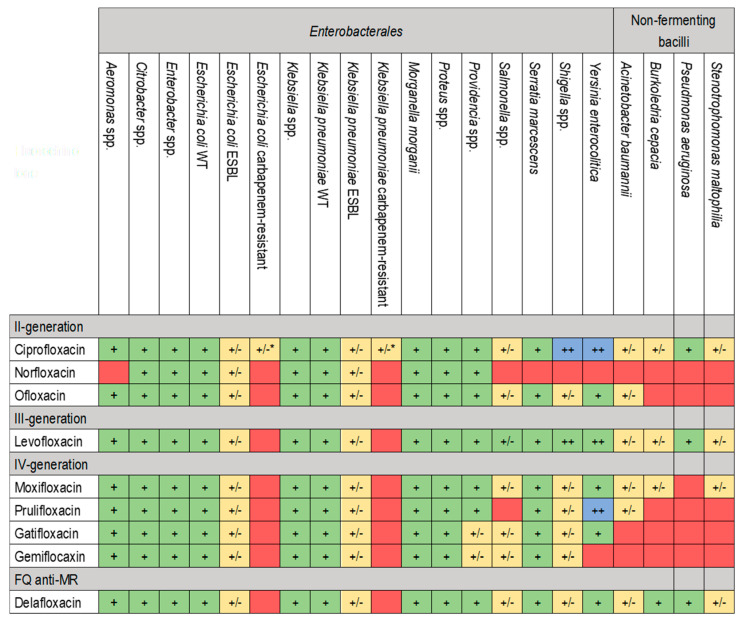
*Enterobacterales* and non-fermenting Gram-negative bacilli fluoroquinolones spectrum. In blue, the agent is recommended for the treatment; in green, the agent is active in vitro, and clinical studies have confirmed its activity against the microorganism; in yellow, the agent has limited or variable activity against the bacteria; in red, the agent is not active. ++: recommended, +: susceptible, +/-: limited utility, FQ: fluoroquinolone, ESBL: extended-spectrum beta-lactamases and WT: wild-type. * Only for some species.

**Figure 3:**
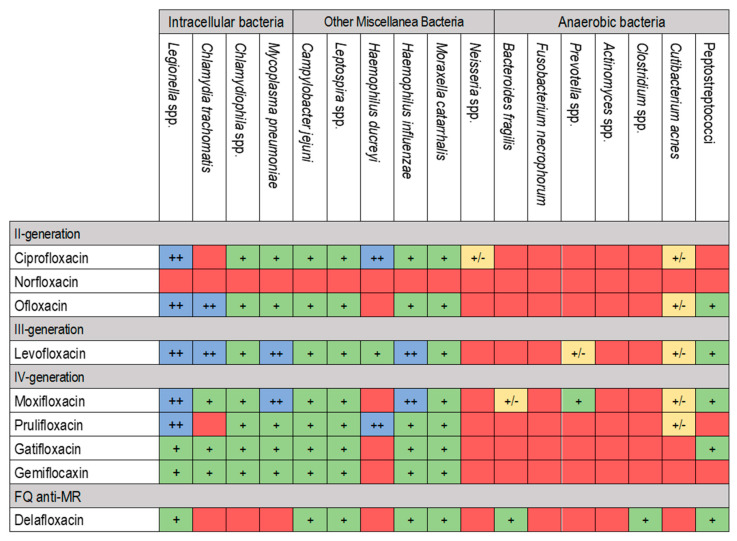
Other microorganisms’ fluoroquinolones spectrum. In blue, the agent is recommended for the treatment; in green, the agent is active in vitro, and clinical studies have confirmed its activity against the microorganism; in yellow, the agent has limited or variable activity against the bacteria; in red, the agent is not active. ++: recommended, +: susceptible, +/-: limited utility and WT: wild-type.

Quinolones are classified into four generations based on their structure and improved spectrum of activity against anaerobic bacteria. Nalidixic acid is a first-generation quinolone with a unique molecular structure compared to other FQs [14,20]. It has an N atom in position 8, while others have a C atom in the same position. From the second generation onwards, an F atom was added at position 6, conferring them a better spectrum of activity, improving the PK/PD proprieties and giving the name FQs. Second-generation FQs (including norfloxacin, ciprofloxacin and ofloxacin) do not have bactericidal activity against anaerobic bacteria. Still, they have an activity against Gram-negative and Gram-positive pathogens, as well as against Mycobacterium tuberculosis [14,18,20]. This issue was resolved for the third-generation FQs like levofloxacin, which has a selected activity against some anaerobic bacteria. The fourth-generation FQs, including moxifloxacin, have an even greater activity against anaerobic bacteria and *Streptococcus pneumoniae* [14,18,21]. This greater activity is also evidenced against M. tuberculosis, as highlighted in different studies, to the point that it is one of the most effective treatments whenever first-line therapy is not feasible [22,23]. In recent years, a new FQ was developed and approved for treating acute bacterial skin and skin structure infections (ABSSSIs) and community-acquired pneumonia (CAP). Delafloxacin is an FQ with in vitro activity against a broad range of Gram-positive and Gram-negative bacteria, anaerobes, *Neisseria gonorrhoeae*, atypical respiratory pathogens and unique spectra against Methicillin-resistant *S. aureus* (MRSA) [24,25]. Figure 2 and Figure 3 report the antimicrobial spectra of Gram-negative and other microorganisms (i.e., intracellular bacteria, anaerobic bacteria, and other pathogens of interest) of different FQs.

Regarding the mode of administration, FQs have been formulated for both oral and intravenous use. They are mainly prescribed for respiratory, gastrointestinal and urinary tract infections due to their excellent distribution in tissues and body fluids [26,27,28]. There is also a topical formulation for eye, skin and soft tissue infections. As with other lipophilic antibiotics, FQs are well absorbed orally and have good bioavailability, ranging from 70% to 99%, depending on the specific FQs [29]. They distribute well in various body fluids and tissues, with the newer FQs reaching serum concentrations higher than that needed to achieve the bactericidal effect [29].

They are usually metabolized in the liver, but some molecules, like ciprofloxacin and levofloxacin, are excreted in the urine unchanged [27,28]. Moxifloxacin has a significant hepatic metabolism, leading to hepatic elimination, thus not usually suitable for urinary tract infections [30].

**Figure 4:**
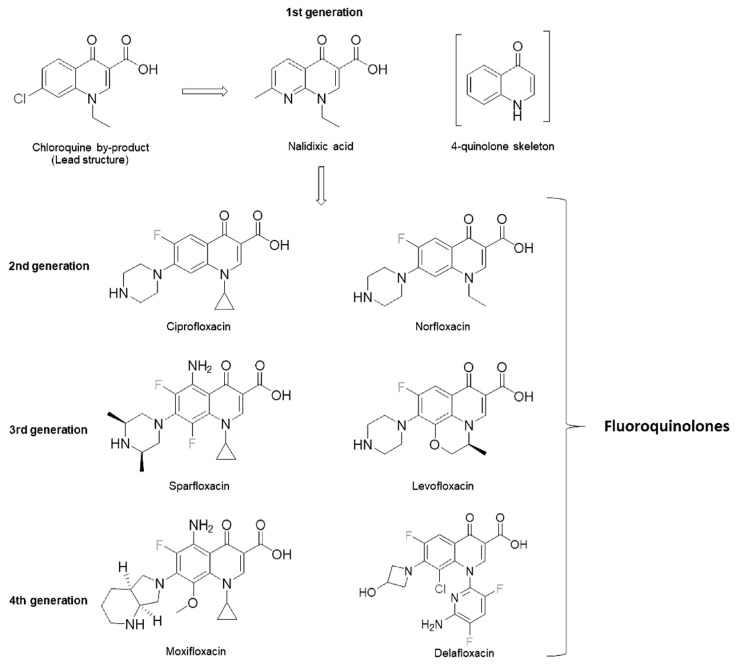
Chemical structure of fluoroquinolones and their division in classes.

Their half-life time permits once- or twice-daily dosing, making them particularly attractive for patients. However, some adverse effects must be considered before prescription, like the risk of tendon rupture, prolonged QT and arrhythmia [20,28,31]. Also, FQs have other common side effects like headache, dizziness or gastrointestinal disturbances that need to be considered. In addition, several drug interactions have been described. Ciprofloxacin inhibits the cytochrome P450 1A2 enzyme (CYP1A2), leading to increased plasma levels of the other drugs metabolized by the same enzyme, like warfarin, amiodarone or tricyclic antidepressants. Also, FQs can regulate a transporter protein, called the P-glycoprotein, involved in the gastrointestinal tract. Thus, if taken with FQs, some immunosuppressants like cyclosporine may have an altered bioavailability [20,32]. FQs and their chemical structure are summarized in Figure 4.

## 3. Activity Against Biofilm and Microbiology

The bacterial biofilm is an architectural colony of microorganisms frequently found in nature. Biofilms are structured microbial communities encased within an extracellular matrix (ECM) that confers significant advantages to their survival, including protection against antimicrobial agents and immune system clearance [33]. The ECM is composed primarily of polysaccharides, proteins and extracellular DNA (eDNA), which provide structural stability and create a physical barrier that reduces the effective concentration of antimicrobial agents. Additionally, the biofilm environment promotes metabolic dormancy and phenotypic heterogeneity among the bacterial population, which are key factors in antimicrobial tolerance. This dormancy limits the efficacy of antibiotics that target metabolically active cells. Gradients of oxygen and nutrients within the biofilm further drive bacterial adaptations, enhancing survival under hostile conditions [5,33,34]. Genetic resistance mechanisms are also often upregulated, including mutations in topoisomerases and the activation of efflux pumps, adding another layer of resilience to the bacterial cells [33].

In medicine, bacterial biofilm has typically been associated with device-related infections, particularly catheter-associated urinary tract infections, breast implants, joint prostheses, mechanical heart valves, ventricular shunts, pacemakers and defibrillators and ventricular-assisted devices [5,33,35]. However, biofilm could also be found in chronic and acute infections, such as dental plaque, cystic fibrosis, otitis media, native valve endocarditis, tonsillitis, necrotizing fasciitis, wounds, osteomyelitis, infective kidney stones, bacterial vaginitis and bladder infections [36].

The cause of device-associated biofilm formatting infections is associated with microorganisms in complex communities that adhere to and grow on device surfaces. Biofilms can consist of single or multiple species, depending on the type and the time of the infections [36]. Common microorganisms found in medical device-associated infections are *S. aureus* and *Staphylococcus epidermidis*. However, multidrug-resistant Gram-negative bacteria, such as *E.coli*, *Klebsiella pneumoniae*, *P. aeruginosa* and *Acinetobacter baumannii*, can be involved in complex device-related infections [36,37].

**Figure 5:**
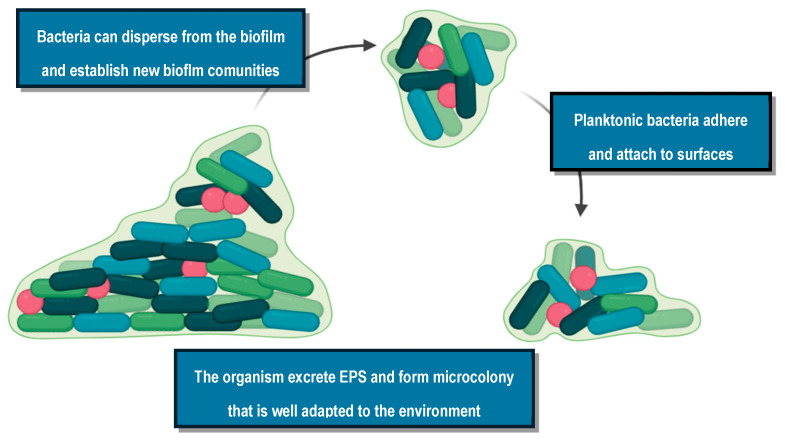
Lifecycle of biofilm formation. EPS: exopolysaccharides.

Research regarding the structure and function of the bacterial biofilm revealed a multistep and complex development. Initially, planktonic cells are attached to a surface, biotic or abiotic. The attached cells start to replicate and develop microcolonies. Bacteria start producing extracellular matrix and DNA, developing a mature biofilm. The last step is biofilm dispersal, which leads to other complications [35]. In Figure 5, we report the life cycle of biofilm formation.

Due to the thick structure and usually a multispecies involvement, eradicating biofilms proves difficult. Several methods have been described: nanoparticles, phage therapy, antisense peptide nucleic acids, quorum sensing inhibitors or enzymatic degradation [38,39].

The therapeutic management on biofilm-related infections depends on the timing of development. For example, if the clinical manifestations appear <6 weeks after implantation of a device, the infection is defined as “early”, and the biofilm is considered immature [40]. If the clinical features manifest >6 weeks, the infection is defined as “chronic”, and the biofilm reaches complete formation (“mature biofilm”). It is possible to retain the device when the biofilm is immature, whereas, with mature biofilm, removing the infection followed by appropriate antibiotic therapy is essential [41].

FQs have been used in different experiments to eradicate bacteria biofilms. Ciprofloxacin was one of the first FQs used against the bacterial biofilm. In a 2002 study, the authors evaluated the use of ciprofloxacin against *S. aureus*, *E. coli* and *P. aeruginosa* [42]. Their results showed that the antibiotic was efficient against planktonic cells, but the cells embedded in the biofilm were not efficiently affected [42]. Similar results were obtained by using levofloxacin in the eradication of *Helicobacter pylori*. In a study from 2014, the authors found that *H. pylori* biofilms exhibited significant resistance to different antibiotics, including levofloxacin; thus, the recurrences of *H. pylori* were possible [43]. Ciprofloxacin and norfloxacin were also used against *Proteus mirabilis* biofilms in an article by Prezekwas et al. [44]. The authors found that both antibiotics inhibited biofilm formation, particularly at high concentrations [44]. The activity of moxifloxacin was also evaluated against the bacteria biofilm produced by *S. aureus*, and the antibiotics showed a significant effect in reducing the total biofilm biomass [45]. However, direct experimental evidence of a cause-and-effect relationship between biofilm characteristics and antibiotics’ lack of efficacy is not always well documented [46]. Based on these studies, it seems that FQs display an impact against the bacterial biofilm, depending on the specific molecule used. However, due to their mechanism of action, FQs have the potential to tackle a wide variety of microorganisms and their biofilm.

## 4. Central Nervous System, Eyes and Ear Infections

Central nervous system (CNS) infections can be divided into community-acquired and healthcare-associated, considering their starting setting. In the latter, we find healthcare-associated meningitis and ventriculitis [47]. This division is important, because the etiology and pathogenic mechanisms differ from those of the community-acquired. In the clinical settings of healthcare-associated meningitis and ventriculitis, there are specific circumstances associated with their development: (1) cerebrospinal fluid (CSF) shunts, (2) CSF drains, (3) intrathecal infusion pumps and (4) deep brain stimulation hardware. All these scenarios imply the presence of external and internal devices, which can be an entrance door for bacteria or are susceptible to colonization of human skin flora. 

Implant-associated infections occur in about 3–15% of cases, which is 4–17% for CFS shunts, 2–22% for external ventricular CFS drainages (EVD) [48,49], 6–20% for intrathecal infusion pumps [50] and 0.62–14.3% for deep brain stimulation hardware [47]. The primary pathogens involved are *S. aureus*, coagulase-negative staphylococci (CoNS), *Cutibacterium*/*Propionibacterium acnes*, *Enterobacterales* and non-fermenting Gram-negatives, including also *A. baumannii* [41,47,51]. EVD and all neurosurgical devices are often subject to biofilm formation [51]. Considering the site of infections and the germs responsible for them, treating healthcare-associated meningitis and ventriculitis relies on antibiotics that can reach adequate concentrations in the CFS and with appropriate antibiofilm activity. The employment of FQs in these infections is the mainstay of antibiofilm treatment when Gram-negative pathogens are involved [41,51]. Unionized, low-molecular-weight, lipophilic and non-protein bound antibiotics, such as FQs or rifampin, have enhanced CNS penetration regardless of inflammation [52,53]. This penetration is higher than other antibiotics, such as in the case of β-lactams drugs [54]. However, antibiotic treatment does not eliminate the need for device removal/surgery, which is the cornerstone of therapy in the presence of neurosurgical implants [55]. 

**Figure 6:**
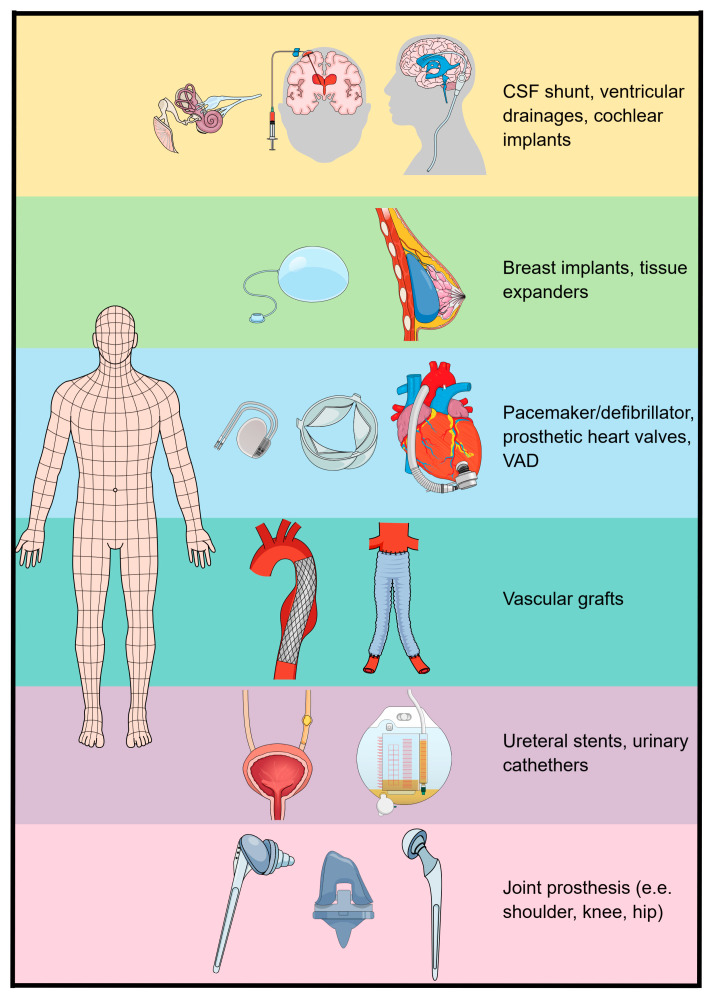
Common implantable medical device locations susceptible to biofilm infections. CNS: central nervous system; VAD: ventricular-assistant device.

The Infectious Diseases Society of America (IDSA) guidelines for healthcare-associated ventriculitis and meningitis indicate ciprofloxacin as one of the essential anti-Gram-negative agents whenever an allergy to β-lactams is documented [47]. Conen et al. agreed with the IDSA and collocated ciprofloxacin as the treatment of choice when treating intra- or extradural infections caused by Gram-negative pathogens [41]. *P. aeruginosa* and other *Enterobacterales* are involved in healthcare-associated ventriculitis and meningitis. Reffuveille et al. evaluated in vitro the ability of ciprofloxacin to decrease the biofilm formed by some strains of *P. aeruginosa* and *E. coli*, demonstrating a straightforward activity of the FQs [56]. In Figure 6, we reported the common implantable medical devices susceptible to biofilm infections.

FQs and other antibiotics, such as trimethoprim-sulfamethoxazole, doxycycline and rifampin, are recommended whenever CNS implants are retained [41]. Intrathecal antibiotic administration, with intravenous infusion, can optimize the bactericidal effect whenever systemic therapy has a poor response or with difficult-to-treat bacteria [41]. Various drugs are recommended for intrathecal infusion, both for Gram-positive and Gram-negative bacteria, such as vancomycin, aminoglycosides, tigecycline and colistin [57].

More studies are needed concerning antibiotics’ effect on biofilm in healthcare-associated meningitis and ventriculitis. Also, it is crucial to make progress in finding solutions for FQs coupled with other agents to enhance the activity of this class of antibiotics against biofilm. In this scenario, nitroxide hybrids are evolving with promising results [58], but it is necessary to take the next step by applying these compounds in real-life studies.

Ocular infections involve different eye districts and are defined as bacterial conjunctivitis, keratitis, endophthalmitis, blepharitis, pre-septal and orbital cellulitis and dacryocystitis [59]. Usually, eye infections are associated with risk factors such as contact lenses, ocular devices, surgery, trauma, age and previous eye disease [59]. The conjunctiva and cornea have been historically considered sterile environments; however, recent research has shown the presence of a microbiome. This microbiome is composed of viruses, fungi and bacteria, mainly represented by *Staphylococcus* spp., *Corynebacterium* spp., *Bacillus* spp. and *Pseudomonas* spp., which is fundamental for ocular health [60,61,62].

The etiology of ocular infections varies between involved districts. CoNS, Streptococci and *Bacillus* spp. (especially if the causative agent is trauma) often cause endophthalmitis [63]. Bacterial conjunctivitis is caused mainly by *S. aureus* and less frequently by Gram-negative bacteria such as *Serratia marcescens* and *P. aeruginosa* [64]. As with other body parts, these infections can also relate to biofilm formation. The most common microorganisms involved in eye-related biofilms are *S. epidermidis*, other CoNS, *P. aeruginosa*, *S. aureus* and *Fusarium* spp., which are responsible for keratitis and endophthalmitis [59,65,66]. The fundamental issue of biofilm infection is the use of contact lenses, which poses a significant concern, even when the storage case surfaces are exposed [59]. The microorganisms responsible for contact lens biofilm-associated infections are mainly caused by *P. aeruginosa*, *S. marcescens*, *S. aureus* and Gram-negative bacteria [59,65,66].

The treatment of ocular infections usually consists of topical and often also systemic antibiotic therapy. Considering the epidemiology cited above, choosing drugs with a broad spectrum coverage is essential, covering both Gram-positive and Gram-negative bacteria. Occasionally, an effective treatment is also a surgical intervention, especially when biofilm is involved [67]. The use of FQs-base ophthalmic solutions has been favored for treating ocular infections due to the broad-spectrum activity and the excellent tissue penetration of these drugs [68]. In 1995, Marone et al. just evaluated the activity of various compounds, including ofloxacin, against CoNS, *S. aureus* and *P. aeruginosa* strains obtained from patients with ocular infections. According to this research, ofloxacin exhibited excellent activity against these germs [69]. In another study, moxifloxacin was compared to chloramphenicol in vitro to evaluate their activity against strains of *S. aureus*, *S. epidermidis*, *P. aeruginosa* and *E. coli*. In this research, moxifloxacin had a better bactericidal activity and capacity to inhibit biofilm formation or disrupt mature biofilm than chloramphenicol [70]. Furthermore, the toxicity of moxifloxacin eye solution had a lower corneal toxicity profile than chloramphenicol [70]. Diriba et al. assessed the susceptibility of various Gram-positive and Gram-negative strains to different drugs, including ciprofloxacin. The latter was among the most effective antibiotics because of its susceptibility, ranging from 70% to 100% among Gram-positive and Gram-negative groups [71]. Interestingly, the study correlated multidrug resistance (MDR) pathogens and biofilm formation [71]. Thirumalmuthu et al. evidenced the same correlations, specifically in *P. aeruginosa* MDR strains [72].

Various approaches have been studied to prevent biofilm formation and fastidious related infections. Among these, an intraocular lens (IOL) designed to release norfloxacin to prevent postoperative bacterial infections after cataract surgery has been tested in vitro and in rabbit models, and it will be available soon [73].

The available literature agrees with the importance of FQs in ocular infections, but there is still much to evaluate and define. Also, the activity of quinolones against biofilm is well known in infections involving various sites, but little is acknowledged in ocular infection-related diseases.

Ear infections can be divided into two categories: (1) middle ear infections, which can be further classified into acute otitis media, otitis media with effusion and chronic suppurative otitis media [74], and (2) otitis externa, known as “swimmer’s ear” [75]. The onset of otitis media is typically preceded by a viral upper respiratory tract infection that causes Eustachian tube dysfunction, ultimately leading to a bacterial infection [74,76]. The pathogens commonly involved are *S. pneumoniae*, non-typable *Haemophilus influenzae* (NTHi) and *Moraxella catharralis* [77,78,79]. Biofilm formation has been demonstrated in patients with chronic suppurative otitis media, persistent otitis media with effusion and the recurrence of otitis media following antibiotic treatment [80,81,82]. The predominant bacterial pathogens in chronic suppurative otitis media are *S. aureus* and *P. aeruginosa* [83], which can form biofilms alongside other otopathogens. The treatment of choice for acute otitis media is based on β-lactams; however, 13% of cases result in treatment failure, likely due to the role of biofilm formation in these infections [84,85]. Shin et al. tested various antibiotics against different strains of NTHi in vitro to assess their ability to disrupt biofilms. The study found that FQs (tosufloxacin and levofloxacin) exhibited greater efficacy against biofilms than β-lactams, suggesting a more prominent role for these molecules in treating acute otitis media [86]. Similar results were reported earlier by Kaji et al., who highlighted that levofloxacin and gatifloxacin significantly inhibited biofilm formation by NTHi strains compared to β-lactams and macrolide antibiotics. Additionally, gatifloxacin effectively killed β-lactamase-negative ampicillin-resistant NTHi, regardless of biofilm thickness [87].

Ongoing studies are evaluating various combination therapies. Novotny et al. suggested a compound of humanized monoclonal antibody fragments targeting a bacterial DNABII protein combined with ofloxacin to eradicate biofilms formed by NTHi. This combination enhanced the activity of both the monoclonal antibody and ofloxacin, leading to biofilm disruption and showing promise as a future treatment [88]. Khomtchouk et al. tested neutrophil elastase inhibitors in combination with ofloxacin in a mouse model of chronic suppurative otitis media caused by *P. aeruginosa*, a known biofilm former. This combination significantly reduced the disease burden, suggesting a potential therapy for this challenging pathogen [89]. In conclusion, the literature supports using FQs as some of the most effective agents against biofilm-producing pathogens frequently involved in ear infections. Table 1 summarizes the principal infections and risk factors related to biofilm formation. Systematic reviews and randomized clinical trials (RCTs) comparing FQ activity with other compounds, such as rifampin, are lacking.

## 5. Osteoarticular and Prosthetic Joint Infections

Osteoarticular infections encompass a range of conditions involving various infection sites and microorganisms, from native osteomyelitis to device-related infections and complications arising from diabetes. Diabetic foot infections, in particular, may lead to osteomyelitis, presenting unique challenges due to specific microbial profiles and issues with drug penetration. No studies have conclusively compared treatment outcomes for diabetic foot infections based on the presence or absence of biofilm. Moreover, data on the role of quinolones in treating these infections remains inconclusive [90].

Biofilm formation plays a crucial role in the pathogenesis of prosthetic joint infections (PJIs), where microorganisms adhere to the implant surface and form biofilms, shielding them from the host immune response and reducing the efficacy of most antibiotics [91]. In osteoarticular infections, biofilms present particularly challenging conditions for treatment. The biofilms formed on bone tissue and prosthetic materials exhibit a highly robust ECM that traps antimicrobials and protects bacterial cells [5]. The hypoxic and nutrient-deprived environment within osteoarticular biofilms promotes metabolic dormancy, while the ECM provides structural integrity and mechanical stability. These biofilms are also marked by a high level of genetic plasticity, facilitating adaptation and resistance. This microenvironment not only complicates therapeutic interventions but also fosters persistence and chronicity of infections [34]. Effective therapeutic strategies must therefore address both the structural and functional properties of biofilms, such as ECM composition, metabolic dormancy and genetic resistance mechanisms. Innovative approaches, such as targeted, multimodal and combined therapies and advanced delivery systems, are critical for overcoming these barriers [5].

Sonication is used to detect bacteria within these biofilms and diagnose infections in removed hip and knee implants [92]. The primary treatment goals for PJIs include eradicating infection, preventing recurrence and preserving joint function [91]. The selected antibiotics must exhibit bactericidal activity against slow-growing, biofilm-protected bacteria and reach high concentrations in the bone. FQs have been utilized for this purpose. However, FQs, like most antibiotics, generally show reduced activity against biofilm-associated bacteria compared to their planktonic counterparts [93]. European guidelines previously evaluated biofilm infection treatments and suggested a potential role for quinolones in treating Gram-negative biofilm-forming infections when used in combination therapies. However, studies from Hengzhuang et al., cited in these guidelines, did not specifically examine the role of FQs in biofilm-associated infections of the bone [94]. In subsequent analyses, the need for updated guidelines was discussed. Promising new observations in vitro and in vivo (animal studies) on biofilm infection therapy were noted but are still awaiting clinical validation [95].

Preclinical studies have demonstrated synergistic effects against biofilm-forming infections, though only a few studies focus on bone and joint infections. For instance, an in vitro study, the activity of fosfomycin, ciprofloxacin and gentamicin, alone and in combination, was evaluated against *E. coli* and *P. aeruginosa* biofilms. The gentamicin-ciprofloxacin combination showed enhanced synergy against *P. aeruginosa* biofilms [96]. In this study, eight *E. coli* and seven *P. aeruginosa* clinical isolates from PJI patients were analyzed, showing that ciprofloxacin had the highest antibiofilm activity against *E. coli* (minimum biofilm bactericidal concentration [MBBC] = 16 μg/mL) compared to *P. aeruginosa* (MBBC = 512 μg/mL) [96]. Another study investigated the in vitro activity of antibiotics (fleroxacin, ciprofloxacin, aztreonam and co-trimoxazole) against *E. coli* ATCC 25922 in both planktonic and biofilm states. Ciprofloxacin exhibited greater potency against non-growing, adherent *E. coli* than the other drugs tested [97]. For *Enterococcus faecalis*, the formation of biofilm and antimicrobial resistance complicates treatment. One study investigated the effectiveness of ciprofloxacin or linezolid, each combined with rifampicin, against *E. faecalis* biofilms. Ciprofloxacin and rifampicin showed the highest potency in reducing biofilm colony-forming units (CFUs) on plastic and bone cement surfaces. Despite this, none of the tested antibiotics completely eradicated biofilms formed within 24 h [98]. Similarly, a retrospective study analyzed the moxifloxacin-rifampin combination for treating PJIs caused by enterococci, streptococci, cutibacteria or polymicrobial infections (47.8%, in which *S. aureus* was involved in the majority of cases), reporting positive results but attributing the antibiofilm effect primarily to rifampicin rather than moxifloxacin [99]. Recent investigations focused on biofilm eradication against *S. aureus* isolates from bone and joint infections, using combinations like moxifloxacin-rifampin and doxycycline-rifampin. These combinations eradicated biofilms in a third of the strains. In contrast, the doxycycline-moxifloxacin combination only inhibited biofilm in a minority of strains, reinforcing that moxifloxacin alone has limited antibiofilm activity [100].

In an animal model focused on *S. aureus* biofilm infections, 61 euthanized Wistar rats were used to study the effects of various treatments on biofilm-associated infection in the femoral medullary cavity, where each rat had a metal implant to simulate device-related infection. The treatment regimens included moxifloxacin monotherapy, moxifloxacin in combination with rifampin administered for 14 days and two control groups for comparison [101]. This study revealed that combination therapy with moxifloxacin and rifampin significantly reduced microbial counts in bone and soft tissues and, crucially, in biofilm formations [101]. In contrast, monotherapies did not yield comparable reductions in microbial loads, suggesting that moxifloxacin alone may lack robust antibiofilm efficacy in this model [101]. These findings appear to contrast with earlier results from an animal study by the same research group, which explored the effects of moxifloxacin on an implant-associated *S. aureus* infection model [102]. In that study, moxifloxacin demonstrated a substantially reduced microbial count within bone, soft tissue, and biofilm compared to vancomycin [102].

The discrepancy between these studies raises questions regarding the standalone efficacy of moxifloxacin against biofilm-associated infections in bone, particularly in monotherapy. In a distinct experimental setting, an infection model involving a metal prosthesis was treated using a novel combination of ciprofloxacin-loaded temperature-sensitive liposomes (TSLs) activated by alternating magnetic fields (AMFs). Here, AMF heating facilitated biofilm disruption while simultaneously triggering the release of ciprofloxacin from the TSL, resulting in a 3-log reduction in CFU of *P. aeruginosa* within the biofilm [103]. Additionally, another study examined the potential of biodegradable ofloxacin-loaded microspheres for biofilm eradication in bone-associated infections. However, the reported data primarily focused on formulation and in vitro biodegradation evaluation [104].

Furthermore, bone cement loaded with levofloxacin demonstrated antimicrobial efficacy against both planktonic and biofilm forms of *S. aureus* when tested in a flow chamber system, effectively targeting extracellular and biofilm-embedded bacteria [105]. This material has also been examined in alternative contexts. Studies on well-established antibiotic-loaded acrylic bone cement showed enhanced levofloxacin release and delayed *S. aureus* biofilm formation while maintaining essential mechanical integrity and biocompatibility properties. However, the clinical significance of these findings is still debated [106].

Emerging materials have also been investigated for biofilm-targeted therapies, particularly FQ conjugates, which combine antibiotics with other media to treat biofilm-forming infections. Ciprofloxacin, moxifloxacin, sitafloxacin and nemonoxacin, along with bisphosphonate-conjugated versions of these antibiotics, were tested and shown to inhibit *S. aureus* biofilms in a dose-dependent manner [107]. In related research, two bone-targeted bisphosphonate-conjugated antibiotics (BCAs)—bisphosphonate-conjugated sitafloxacin (BCS) and hydroxybisphosphonate-conjugated sitafloxacin (HBCS)—were assessed within infected osteocyte-lacuno-canalicular networks (OLCNs). Initial findings indicate that these BCAs achieved targeted *S. aureus* eradication within the OLCN, supporting BCAs as a promising strategy to overcome the biodistribution limitations of conventional antibiotic delivery. Future research is anticipated to further validate bacterial phenotypes in the OLCN of *S. aureus*-infected bones in animal models treated with BCS and HBCS [108]. The potential for clinical application of these conjugates is now an active consideration. A recent study evaluated ciprofloxacin-loaded calcium carbonate (Cip-loaded CaCO_3_) nanoparticles for their biocompatibility and antibacterial/antibiofilm efficacy against common pathogens in osteomyelitis. The nanoparticles demonstrated favorable in vitro compatibility with human red blood cells, significant antimicrobial activity and low cytotoxicity, suggesting the potential for further application in bone infection models [109].

In conclusion, most studies investigating antibiofilm efficacy in bone infections remain limited by methodological challenges inherent to this research area. Traditional approaches—often focused on evaluating the impact of intravenous or oral antibiotic administration on biofilm formation—face significant limitations when applied to complex bone environments, where achieving therapeutic drug concentrations is difficult and bacterial biofilms present unique barriers to effective treatment. Recent research trends indicate a shift towards local antibiotic delivery systems as a promising strategy to overcome these limitations. By delivering antibiotics directly to the site of infection, these systems, including antibiotic-loaded bone cement, nanoparticles and bisphosphonate-conjugated antibiotics, aim to achieve higher local drug concentrations, enhance biofilm disruption and potentially improve clinical outcomes in bone infections like osteomyelitis. While still in the experimental phase, this approach represents an evolving paradigm that prioritizes targeted and sustained antibiotic exposure within infected bone tissue, warranting further investigation to establish optimized, clinically applicable strategies for combating biofilm-associated bone infections.

## 6. Vascular Prosthetic Infections and Endocarditis

The use of synthetic material for reconstructive vascular surgery was first reported during the early 1950s. Infection involving vascular graft prostheses is a devastating complication of reconstructive vascular graft surgery and is associated with high morbidity and mortality rates of up to 75% [110]. The frequency of vascular graft infections (VGIs) can depend on the anatomic location of the graft prostheses, ranging from a rate of 1.5–2% for most extra-cavitary grafts to 6% for vascular grafts in the groin [110,111]. VGIs are most common after emergency urgent procedures and after reoperation [110]. The microbiology leading VGIs has changed over the years. Historically, *S. aureus* was the predominant microorganism in VGIs [112]. Gram-positive bacteria, including *S. aureus*, CoNs and *Enterococcus* spp., cause up to 58% of VGIs [113]. However, due to the changes in the patient’s characteristics and surgical procedure, new pathogens have been involved in VGIs, such as MDR Gram-negative microorganisms, polymicrobial infection and *Candida* spp. [110]. Remarkably, *P. aeruginosa* is now the most frequent Gram-negative pathogen involved in VGIs [114].

Intraoperative bacterial contamination of the vascular graft is considered the most common cause of VGIs. Less common causes of VGIs are the spread of infection from a contiguous site or bacteremia [110,115]. Like other chronic and devices-related diseases, biofilms can be crucial in VGIs. Biofilms resist antibiotics mainly by preventing them from reaching the bacterial cells in the biofilm matrix and limiting their efficacy [116]. In addition to their antibiotic resistance, biofilms protect the bacteria against the innate immune system and phagocytosis, creating an anatomical reservoir for the infection [117].

The best management for VGIs is not always straightforward. It depends on the location of the graft, the extent of the infection, clinical presentation, the bacteria involved and the patient’s comorbidities [110,111]. The resolutive management of prosthetic VGIs is usually made by excision of the graft, complete debridement of the infected surrounding tissues, restoration of blood flow distal to the infected graft and appropriate antibiotic therapy [118]. In cases where a conservative approach is taken, chronic suppressive antibiotic therapy is often considered [118]. An adequate antibiotic regimen should have the following characteristics to treat VGIs: (i) bactericidal activity in the bacteria growth phases; (ii) reduction of the microbial burden; (iii) penetration within the biofilm; (iv) prevention of further biofilm formation [118].

The in vitro role of FQs seems to have adequate characteristics to contrast with biofilm formation. For example, data from the experimental model showed how ciprofloxacin had excellent activity in reducing biofilms formed by clinical methicillin-susceptible *S. aureus* (MSSA) strains on the surface of biological and synthetic vascular grafts [119]. Notably, in the case of suppressive treatment, FQs can play a crucial role in the patient’s chronic management. Despite the possible risk of vascular aneurysms linked to these antibiotics [120], the association of rifampicin and FQs could be preferred in proven cases of MSSA VGIs and FQs alone in proven cases of *Enterobacterales*. The benefits of FQ-based chronic suppression therapy seem more significant in infections with an appalling short-term prognosis [121]. FQs could also be a reasonable oral step down therapy in case of Enterococci infections. Patients were treated mainly with an antibiotic combination containing FQs with or without rifampicin, which had a good effect on long-term prognosis. That choice was primarily performed in case of polymicrobial infections, taking advantage of the proprieties’ FQs (high tissue concentrations and antibacterial activity in biofilms and broad-spectrum activity) [122].

New possible strategies were presented with FQs usage as local agents in device-related materials. For example, levofloxacin was used in an albumin-sealed Dacron graft to prevent VGIs caused by *S. aureus* [123].

Despite recent advances in antimicrobial and surgical therapy, infective endocarditis (IE) remains a significant clinical problem, with an attributable mortality rate of 20–25% [124,125]. IE is a life-threatening condition that can be verified on the native valves (NVEs) and prosthetic valves (PVEs), endocardial surface or indwelling cardiac device [126]. Endocardial tissue represents an optimal reservoir for bacteria, which develop an endocardial biofilm, which describes complex communities embedded into a matrix of secreted macromolecules [108]. Mature vegetations comprise an amalgamation of inflammatory cells, fibrin, platelets and erythrocyte debris. The initial platelet–fibrin clot provides a nidus for bacterial adherence, furthers platelet aggregation and facilitates biofilm formation [127]. Microbiological isolates can differ significantly in the different IE settings. *S. aureus* is associated with NVEs in 27% of the cases, followed by streptococci (26%) and enterococci (12%) [126]. Staphylococci are considerably more prevalent in PVEs (32%), followed by streptococci (25%) and enterococci (16%) [126]. In cardiac device-related infective endocarditis patients, the majority of the infections are caused by staphylococci (54%, CoNs 25.2%), followed by streptococci (12%) and enterococci (5%) [126].

The management of IE patients consists of medical and surgery therapy. FQs do not represent the primary treatment in the case of IE, excluding particular scenarios where ciprofloxacin can be an alternative to the standard of care (i.e., Gram-negative IE) [128]. However, due to their optimal oral bio-dispensability, FQs can be a reasonable oral step down therapy. Notably, in the POET trial, the FQs combined with a second agent were used in several cases of *S. aureus*, *E. faecalis*, CoNs and streptococcal IE [129]. It is important to note that the oral de-escalation strategy, also with FQs, was not inferior according to the 6-month composite outcome of mortality, unplanned cardiac surgery, embolic events or relapse; these results were confirmed during an extended follow-up after three and five years [129,130,131]. In the literature, few studies were conducted explicitly on FQs’ efficacy in the case of IE. However, some authors suggested that quinolones diffuse rapidly into vegetation; thus, a rapid response is anticipated [132], which may affect FQs favorably. However, in the literature, few clinical data are present. Unfortunately, a general recommendation of FQs in treating IE cannot be made. Large-scale RCTs or meta-analyses that evaluate the role of FQs in VGIs and IE are not available in the literature. Table 2 summarizes the FQs’ role in managing VGIs and IE.

## 7. Pulmonary Infections

Pneumonia is one of the leading causes of death and morbidity worldwide [133]. It could be complicated by biofilm formation, which could contribute to the persistence and severity of the disease due to the protection of bacteria from the immune system and antibiotic treatment. The biofilm in pneumonia is commonly due to *P. aeruginosa*, *S. aureus* and *K. pneumonia*, especially in hospital settings [134]. Predisposing conditions that could increase the probability of biofilm formation include chronic obstructive pneumonia disease (COPD) and cystic fibrosis [36,135,136,137,138]. Lung biofilm formation is more common in the intensive care unit (ICU), especially in people with ventilator-associated pneumonia (VAP), due to medical devices such as endotracheal tubes [139,140,141,142]. It is well known that, a few hours after intubation, the endotracheal tube starts being colonized by microorganisms [143] that form a biofilm on its surface, especially in the interior of the distal part [144,145]. In the case of VAP, it is difficult to discriminate if the colonized microorganism is causing the infection. The multidrug resistance ESKAPE pathogens (*E. faecalis*, *S. aureus*, *K. pneumoniae*, *A. baumannii*, *P. aeruginosa* and *Enterobacter* spp.) significantly contribute to VAP etiology.

On the contrary, microorganisms of the normal oral flora, which generally start the formation of biofilm, are rarely involved in VAP development [139,146]. It is also fundamental to the timing of VAP development; some studies show that, if it develops a few days (2–5) after intubation, it is likely caused by antibiotic-sensitive bacteria such as MSSA; on the other hand, if it is developed after five days, it frequently involves multidrug-resistant microorganism like MRSA, *P. aeruginosa* difficult-to-treat (DTR) and carbapenem-resistant *Enterobacterales* (CRE) [143,147]. There are many strategies to prevent VAP, but these are not the focus of this review [148,149].

Regarding the treatment of VAP, it is essential to remember that guidelines recommend using narrow-spectrum antibiotics, including levofloxacin and moxifloxacin, in patients with a suspected low risk of resistance and early onset of VAP without septic shock. However, it is mandatory to adapt the empiric treatment according to the local epidemiology [150,151,152]. FQs, such as ciprofloxacin, levofloxacin and moxifloxacin, are commonly used to treat respiratory infections, including pneumonia [153]. These antibiotics have excellent penetration profiles, particularly in the lungs, making them valuable agents in treating pneumonia, including cases complicated by biofilm formation [154,155]. The efficacy of FQs against biofilms, alone or in combination, has been reported in several studies [156,157,158]. This is particularly true in the case of *P. aeruginosa* infections, where Usman et al. found inhibitory effects of ciprofloxacin and levofloxacin against a biofilm-forming *P. aeruginosa* colony. In the same study, the authors showed that FQs, combined with cephalosporins (ciprofloxacin and cefepime), have strong synergistic effects against biofilms [156]. Moreover, quinolones are part of combination therapy schemes suggested by the IDSA guidelines for *S. maltophilia*; its role as a pathogenic agent is not always clear and often presents as part of polymicrobial infections. However, infections by *S. maltophilia* can be severe, and its treatment is particularly challenging due to biofilm formation, antimicrobial resistance and lack of shared breakpoints for most antibiotics [159].

Regarding the new FQs, only delafloxacin and zabofloxacin are approved for CAP treatment. In particular, zabofloxacin is indicated for acute bacterial exacerbation of COPD. Delafloxacin is approved for treating CAP caused by many pathogens, including *S. pneumoniae*, MSSA, *K. pneumoniae* and *P. aeruginosa* [160]. In addition, delafloxacin has been shown to maintain efficacy in many strains of *P. aeruginosa* and is resistant to levofloxacin in people with cystic fibrosis [161]. Their role in pneumonia is crucial, also thanks to their efficacy against biofilms, which has been extensively proven [162,163,164]. The biofilm inhibition caused by delafloxacin on different *S. aureus* strains was also tested during an in vitro study. Delafloxacin was an effective antibiotic, obtaining a reduction of the bacterial viable count by more than 50%, with a biofilm penetration capacity that can vary between 0.6% and 52% [163,164].

## 8. Reproductive and Urinary Tract Infections

Urinary tract infections (UTIs) include lower and upper urinary tract infections. Among other risk factors, indwelling urinary catheters, stents and other devices and the presence of lithiasis are of interest for our review, being predisposing factors for biofilm formation. Moreover, the most frequent uropathogens account for biofilm formation, among other virulence factors: *E. coli*, *Klebsiella* spp., *Proteus* spp., *P. aeruginosa* and *Staphylococcus* spp. [165,166,167]. Uropathogenic *E. coli* (UPEC) is the most common microorganism implied in UTIs (90% of the isolates) [168]; among these, biofilm formation accounts for 80% of community-acquired UTIs and 65% of nosocomial [169]. Biofilm is recognized as responsible for complicated and recurrent UTIs [170]. Moreover, higher biofilm formation is connected to the production of virulence genes [171] and horizontal transfer or resistance genes [172]. *P. mirabilis* is known to form biofilms on a variety of living and non-living surfaces [173]; biofilm formation in UTIs is the one that has been better studied. It can lead to catheter encrustation and blockage, ascending UTIs and lithiasis [174]. As suggested for UPEC, biofilm formation of *Klebsiella* spp. is related to enhanced antimicrobial resistance. Although sensitive strains and resistant strains can produce biofilm but cannot produce biofilm, several studies have shown a higher prevalence of antimicrobial resistance among biofilm-producing strains and higher-level biofilm production among MDR strains [175,176]. Regarding *Proteus* spp., Przekwas et al. evaluated the impact of FQs against Proteus mirabilis isolated from UTIs and their effect against biofilm. The study showed that the tested strains inhibited biofilm formation by ciprofloxacin and norfloxacin. Moreover, the biofilm reduction rate was correlated with the increasing concentration of FQs [44]. For *P. aeruginosa*, biofilm formation is among the numerous resistance mechanisms that make this pathogen an alarming threat and a primary target for new treatment strategies, as advocated by the World Health Organization (WHO) [177,178]. Despite being mostly considered saprophytes, staphylococci are increasingly recognized as causative agents of UTIs. Biofilm formation is one of the underlying pathogenic mechanisms responsible for the majority of Staphylococcal-related UTIs. A recently published systematic review reported a pooled prevalence in UTIs of *S. aureus* and CoNs of 8.71% (95%CI: 6.145–11.69) and 13.17% (95%CI: 8.08–19.27), respectively. Moreover, 88 *S. aureus* strains were biofilm producers, of which 35% were moderate and 48% were strong [179].

As mentioned, biofilm formation favors anatomical site colonization and undermines the antimicrobial treatment efficacy. FQs are effective against most uropathogens, and their pharmacodynamics are favorable for their use in the setting of UTIs. Although there are increasing reports of biofilm-producing quinolone-resistant strains [180], this class has also been proven to be a valuable option against biofilm-forming strains. Moreover, increasing data on extended-spectrum beta-lactamases (ESBL) microorganism FQ-resistant strains are emerging worldwide [181]. However, data from the literature suggest that FQs are good antibiofilm agents. The study by Elhosseini et al. demonstrated the activity of ciprofloxacin against *P. mirabilis*, especially against biofilm both in vivo and in vitro [182]; this is coherent with what was reported in previous studies focusing on UTIs [183]. Also, Whelan et al. proved the efficacy of ciprofloxacin against biofilm-forming strains of UPEC (52.6% of the strains included); however, the authors highlighted the risk of forming stronger biofilms when exposed to a subinhibitory concentration of the antibiotic [184]. Similar data were reported in other real-life studies [185]. Besides highlighting the risk of subinhibitory concentrations of ciprofloxacin, Rafaque and colleagues supported the activity of levofloxacin against biofilm-forming UPEC [186].

Regarding combination therapy, the in vitro data by Slade-Vitković et al. showed a higher inhibition of biofilm formation in *P. aeruginosa* strains with fosfomycin in combination with ciprofloxacin vs. ciprofloxacin alone [187]. The combination with azithromycin has also shown promising results against biofilm-producing strains of *P. aeruginosa* [188]. Regarding levofloxacin, its in vitro combination with azithromycin showed no benefit, while the in vivo data were promising [189].

Innovative strategies, including FQs combined nanoparticles/nanocarriers DNase applied to UTIs, also show optimistic data, with other antibiofilm components boosting their ability to inhibit biofilms [190,191]. 

In conclusion, FQs should be used cautiously when facing potentially biofilm-producing microorganisms due to the risk of enhancing biofilm production when exposed to a subinhibitory concentration. However, especially with the introduction of innovative strategies, this class remains a valuable option for empiric and target antimicrobial therapy of UTIs. 

Regarding genital tract infections, biofilm is vital for the adhesion and survival of several causative microorganisms [192]. *Neisseria gonorrhoeae* is known to produce a biofilm, which allows the bacteria to evade host immunity and favor the horizontal transfer of resistance genes [193]. FQs could be an important asset in *N. gonorrhoeae* management due to worldwide increasing resistance to ceftriaxone [194]. Nonetheless, resistance to FQs is also being reported [195,196]; therefore, local epidemiological data are crucial to determine whether FQs could still play a role in managing *N. gonorrhoeae*. Pathogenic mechanisms of *Mycoplasma hominis* and *Mycoplasma genitalium* are less clear. However, both can produce a biofilm [197,198]. As for *N. gonorrhoeae*, resistance to FQs is being reported [199]. Over the past decade, *M. genitalium* has become increasingly resistant to antimicrobials, including macrolides and FQs (~7.7%) [200]. Their resistance is mediated by mutations in DNA topoisomerase (parC, amino acid positions S83 and D87) and DNA gyrase (gyrA, positions M95 and D99) genes [200].

Similarly, data regarding biofilm in *Ureaplasma* spp. infections are lacking; however, it is also noted as a biofilm-forming microorganism [201]. In a recent systematic review, the proportions of ciprofloxacin, ofloxacin, moxifloxacin and levofloxacin resistance in *Mycoplasma* and *Ureaplasma* urogenital isolates were problematic, with a percentage of resistance reported that varied from 59.8% (ciprofloxacin) to 5.3% (levofloxacin) [202]. These findings represent a worrisome trend in antibiotic resistance in the case of *Mycoplasma* and *Ureaplasma* infections, where FQs were widely used as anti-*Mycoplasma* agents and intracellular sexual bacterial infections when tetracycline and macrolides failed [202].

The role of *Gardnerella vaginalis* is exciting; not only does it play a significant role throughout the production of biofilm, but the same biofilm can function as a scaffold to which other bacteria can adhere and contribute to the pathogenesis of bacterial vaginosis or other genital infections [203]. In these settings, the polymicrobial biofilm represents the main problem in microbiological eradication, and *G. vaginalis*, *Atopobium vaginae* and *Lactobacillus* ssp. sustain it [204]. Some authors, mainly for the potential role of polymicrobial biofilm, extrapolate the possibility of fluoroquinolone usage in bacterial vaginosis. However, the clearance of such a biofilm is far from being achieved; attempts to eradicate *G. vaginalis* biofilm with moxifloxacin had inconsistent results [204]. Table 3 summarizes the FQ’s role in reducing biofilm formation in urinary and genital infections.

## 9. Skin and Soft Tissue Infections

FQs are extensively utilized in treating skin and soft tissue infections (SSTIs) due to their broad-spectrum activity [205] and effective tissue penetration [206]. The penetration rates into the skin are approximately 62–73% for moxifloxacin, 85% for ofloxacin, 94–104% for levofloxacin, 117%, for gatifloxacin and 121% for ciprofloxacin [207]. Although the skin is an accessible site for antibiotic penetrations, many factors can reduce drug distribution, such as reduced vascularization in chronic conditions (i.e., diabetic foot), peripheral vascular diseases and skin abscesses [208,209,210].

Many classifications have been proposed for skin and soft tissue infections. According to the IDSA, we can distinguish infections by three key factors: (i) skin extension, with uncomplicated superficial infections (uSSTIs), and complicated ones (cSSTIs) involving deeper tissues; (ii) rate of progression, categorizing infections as either acute or chronic; and (iii) the presence of tissue necrosis, differentiating between necrotizing and non-necrotizing infections [211].

The role of biofilm in the pathogenesis of chronic skin infections is more widely recognized than in acute infections [212]. In particular, the presence of biofilms in SSTIs as a major virulence factor is well established in pressure ulcers [213], chronic diabetic wounds [214] and surgical site infections [215]. The biofilm in SSTIs poses a substantial therapeutic challenge independently of the multidrug resistance phenotype [216]. Bacteria within these biofilms generate a protective matrix that restricts antibiotic penetration, shields them from immune defenses and leads to metabolic inactivation due to nutrient and gas limitations, frequently resulting in chronic and treatment-resistant infections [214,217]. The bacteria most commonly involved in biofilm formation in skin infections include *S. aureus*, *S. epidermidis*, *P. aeruginosa*, *E. coli* and *E. faecalis* [215,218].

Using FQs for surgical site infections and chronic diabetic wounds is supported by the IDSA guidelines [90,211,219]. The World Society of Emergency Surgery and the Surgical Infection Society Europe consensus raised caution in using FQs for MRSA infections [220]. The European Society of Clinical Microbiology and Infectious Diseases (ESCMID) recommends treatment with two antibiotics with different mechanisms of action in patients with chronic wound infections [94]. Ciprofloxacin, alone or in combination, showed antibiofilm properties, particularly against *P. aeruginosa* [156]. The use of topical antibiotics in combination with systemic antibiotics is controversial for chronic SSTIs. The latest IDSA guidelines suggest not using topical (sponge, cream and cement) antibiotics in combination with systemic antibiotics for treating either soft tissue infections or osteomyelitis of the foot in diabetic patients [219]. Given this premise, a ciprofloxacin-based chitosan (CS)-hydrolyzed starch nanocomposite has shown promising antibiofilm properties in in vitro and in vivo models [221]. Moxifloxacin is extensively used in SSTI treatment. Its use has proved effective in four clinical trials: three focused on SSTIs [222,223,224] and one on diabetic foot infections [225]. Moxifloxacin is effective against biofilm-associated infections, particularly those involving a mature MRSA biofilm [163].

There is limited evidence of levofloxacin in the context of biofilm-related infections.

Delafloxacin, a newer FQ, has shown notable efficacy in skin and soft tissue biofilm-associated infections, primarily due to its high tissue penetration and sustained activity in acidic environments typical of chronic wound sites. This unique activity profile under acidic conditions enhances its effectiveness, specifically against biofilm-producing pathogens, including MRSA [24,163,164].

A recent comparative study of different FQs and their activity on biofilm-producing isolates was conducted. Ribeiro et al. found out that delafloxacin was the most active FQ against Staphylococci (including MRSA) and *P. aeruginosa* when compared to other FQs, such as ciprofloxacin and levofloxacin [226]. Unfortunately, the study did not directly evaluate the antibiofilm activity of the different drugs. Therefore, further studies on this topic are warranted.

## 10. Digestive Infections

Mucosal biofilms are often considered early indicators of disease progression and have been associated with irritable bowel syndrome, inflammatory bowel diseases (IBD) and gastric and colorectal cancers [227]. Furthermore, it has been demonstrated that biofilm composition can influence the course of IBD [228]. For instance, macroscopically visible mucosal biofilms have been identified in 57% of IBD patients, 34% of ulcerative colitis patients and 22% of Crohn’s disease patients compared to only 6% of healthy individuals. Additionally, mucus-invasive colonic biofilms are more prevalent in colorectal cancer patients (50% of colorectal cancer patients compared to 13% of healthy individuals) [229,230,231].

Adherent-invasive *E. coli*, which has a strong capacity to form biofilms, produces extracellular polymeric substances that enhance bacterial survival [103]. Similarly, the formation of biofilms by *Clostridioides difficile*, *Salmonella* spp. and *Campylobacter* spp. contributes to recurrent infections and is a protective reservoir against antibiotics [232,233,234,235]. Thus, while the data on the association between biofilms and gastrointestinal diseases are increasing, the underlying disease relevance of these biofilms remains to be fully elucidated. In certain conditions, however, the disease relevance is clearer, as is the benefit of antibiofilm antibiotic therapy, specifically with quinolones, which is the focus of this paper. Clinical conditions where antibiofilm therapy is notably beneficial include infected gallstones or biliary stents (+/- cholangitis). Ideally, the removal of stones or stents is the optimum solution for eradicating the infectious focus; however, this is not always feasible. In such cases, medical therapy should target biofilm activity. Although the benefit of antibiofilm activity of FQs against these chronic/recurrent conditions is a strong point in favor of the use of these drugs, it is important to note that the chronic use of FQs can be related to important side effects and long-term disability (i.e., musculoskeletal disorders) [7]. Moreover, the chronic use of FQs can be associated with a high risk of microbiota damage, disturb the defense system and lead to bacterial antibiotic resistance [236].

Regarding *Enterobacterales* (and *P. aeruginosa*), ciprofloxacin is the most studied antibiotic for biofilm activity within the FQs class. As early as 2000, it was demonstrated that ciprofloxacin, unlike ampicillin, could rapidly penetrate *K. pneumoniae* biofilms [237]. A study published by Aditya et al. in 2021 aimed to characterize the biofilm-forming ability of E. coli under in vitro gut conditions in the presence of ciprofloxacin. While ciprofloxacin effectively eradicates biofilms formed by most isolates, conditions such as low temperature, bile and pH still allow resistance to high ciprofloxacin concentrations [238].

Combinations of antibiotics containing ciprofloxacin have also been investigated for biofilm treatment. For instance, combining ciprofloxacin with cefepime demonstrated synergism, significantly reducing the minimum biofilm inhibitory concentration and the minimum biofilm eradication concentration for *P. aeruginosa* [156]. Similarly, combining ciprofloxacin with ampicillin and tobramycin showed efficacy in eradicating biofilms of two out of four biofilm-producing enteroaggregative E. coli strains tested [239].

Recently, a new photoactivated ciprofloxacin was tested against *S. enterica*, *E. coli* and catheter-derived bile duct microbiomes. Photoactivated drugs offer precise, localized treatment with reduced side effects and enhanced efficacy by being activated only at targeted sites. The authors demonstrated that photoactivated ciprofloxacin effectively prevented biofilm formation and reduced bacterial viability compared to regular ciprofloxacin [240].

It is essential to critically assess these results, especially in cases of mature biofilms, where the antibiofilm effect might become clinically irrelevant.

## 11. Discussion

FQs are broad-spectrum antibiotics with excellent tissue diffusion via oral and intravenous administration. Their PK/PD characteristics allow their use in different human districts, even the ones hard to normally reach, such as the prostate or the CNS. Their versatility in treating Gram-positive and Gram-negative pathogens makes them the treatment of choice for various infections [1,3]. Furthermore, they are made in different formulations to enhance their use.

Eventually, their capacity to eradicate biofilms is well documented in in vitro studies. However, in vivo studies, particularly large-scale RCTs or meta-analyses, are lacking at the moment of the present narrative review. Even though the existent literature puts FQs as one of the most active antibiofilm agents, most of the evidence is extracted from in vitro studies, and there is a need to implement and increase real-life research. Most studies indicate that the antibiofilm activity of FQs is superior to that of beta-lactams and glycopeptides but lower than that of minocycline and fosfomycin. Combinations of FQs with fosfomycin, minocycline, rifampin and aminoglycosides show promising antibiofilm activity. Also, despite their undoubtful usefulness, they are burdened by significant toxicity, especially considering their use in the setting of aged people or infections requiring months of treatment [7]. New research should focus on possible new strategies for FQ use to maximize the antibiofilm activity and reduce the side effects. As highlighted in this review, new compounds are being evaluated in different settings to improve the effectiveness of FQs and avoid unnecessary morbidities. These combined treatments balance the intrinsic characteristics of FQs, giving a “second youth” to this class of antibiotics. At least, newer FQs available in clinical practice show good antibiofilm activity but are evaluated for a few types of infections. Much more is necessary to establish the broadness of their scope.

## 12. Materials and Methods

A comprehensive literature search was conducted to identify relevant studies concerning FQs and biofilm. The search strategy was implemented using online databases (PubMed/MEDLINE/Google Scholar) and books written by experts in microbiology and infectious diseases. The search was not restricted by language, region, study type or publication date and covered articles up to the cutoff date of October 2024. Conference abstracts or unpublished data were excluded. The Boolean operator used was “AND”. The following keywords were used: “Fluoroquinolone AND Biofilm”, “Therapy strategies AND Biofilm”, “Quinolone AND Biofilm”, “Fluoroquinolone AND Central nervous system”, “Fluoroquinolone AND eye infections”, “Fluoroquinolone AND eir infections”, “Biofilm AND Central nervous system infections”, “Fluoroquinolone AND Osteoarticular infections”, Biofilm AND Osteoarticular infections”, “Fluoroquinolone AND Prosthetic joint infections”,” Biofilm AND Prosthetic joint infections”, “Fluoroquinolone AND Spondilodiscitis”, Biofilm AND Spondilodiscitis”,” Fluoroquinolone AND Vascular prosthetic infections”,” Fluoroquinolones AND endocarditis”, “Biofilm AND Vascular prosthetic infections”, “Biofilm AND endocarditis”, “Fluoroquinolone AND Pneumonia”, “Biofilm AND Pneumonia”, “Fluoroquinolone AND Urinary tract infections”, Biofilm and Urinary tract infections”, “Fluroquinolone AND Skin and soft tissue infections”, “Biofilm AND Skin and soft tissue infections”, “Fluoroquinolone AND Gastroenteritis”, “Biofilm AND Gastroenteritis”, ”Systematic review AND Fluoroquinolone” and “Systemic review AND Biofilm. Studies were included in this narrative review if they met the following criteria: studies reporting the in vitro activity of FQs and reviews reporting the in vivo activity of FQs. We screened the articles by title, abstract and full text. After an initial screening of titles and abstracts of the published articles, the reviewers evaluated the full articles to assess the eligibility for each study’s inclusion in this narrative review. A study was included to determine if it was likely to provide valid and valuable information according to the review’s objective.

## 13. Conclusions

Managing infections involving biofilms formed by bacteria remains a significant challenge in clinical practice. FQs maintain a central role in this scenario, but improving our knowledge of the weapons we dispose of is necessary. This review aims to show the reader a complete overview of the activity of FQs in treating infections involving biofilms, underlining their remarkable capacity to do so. Also, we want to bring out what is new in the battle against bacteria capable of biofilm formation, displaying how FQs continue to demand a pivotal role in this setting.

## Figures and Tables

**Table 1:** Central nervous system, eye and ear infections, risk factors for biofilm formation and principal pathogens implicated.

Infection Area	Risk Factors	Pathogens	Biofilm Formation
CNS	Shunts/drains, Intrathecal infusion pumps Deep brain stimulation hardware	*S. aureus*, CoNS, *Cutibacterium acnes*, *Enterobacterales* spp., *A. baumannii*	High risk due to external/internal devices
Eye	Contact lenses Surgery Trauma Ocular devices	*Staphylococcus* spp., *Corynebacterium* spp., *Bacillus* spp., *Pseudomonas* spp.	Common in contact lenses and storage cases
Ear	Viral respiratory infections Eustachian tube dysfunction	*S. pneumoniae*, *Haemophilus influenzae*, *Moraxella catharralis*, *S. aureus*, *P. aeruginosa*	Biofilm formation in chronic/recurrent otitis media and “swimmer” ear

CNS: central nervous system; CoNS: coagulase-negative staphylococci.

**Table 2:** Vascular graft infections and infective endocarditis and the role of fluoroquinolones in their management.

Infection	Microbiology	Management	Role of Fluoroquinolones
VGI	Mostly Gram-positive (*S. aureus*, CoNs, enterococci). Increasing MDR Gram-negatives, *P. aeruginosa*, and candida.	Excision, debridement, and antibiotics. Chronic suppression with antibiotics.	Effective in biofilm reduction. Useful in chronic treatment and certain infections.
IE	*S. aureus*, streptococci, and enterococci.	Medical and surgical therapy.	Used for oral step-down therapy in specific cases. Limited studies on efficacy. Some suggest quick biofilm penetration for treatment.

CoNS: coagulase-negative staphylococci; IE: infective endocarditis; VGI: vascular graft infections.

**Table 3:** Role of FQs in reducing biofilm formation in urinary and genital infections.

Infections	Microorganisms Implicated in Biofilm Formation	Role of Biofilm	Role of Fluoroquinolones
UTIs	*E. coli*, *Klebsiella* spp., *P. aeruginosa*, *Staphylococcus* spp.	Biofilm formation is common in 65–80% of cases. Contributes to recurrent and complicated urinary tract infections.	Effective against most uropathogens. Risk of enhanced biofilm formation at sub-inhibitory concentrations.
Genital Tract Infections	*N. gonorrhoeae*, *Mycoplasma hominis*, *Mycoplasma genitalium*, *Gardnerella vaginalis*	Biofilm protects pathogens from immune system and antibiotics. Facilitates resistance gene transfer.	Useful for *N. gonorrhoeae* treatment, but resistance is emerging. Limited evidence for *Mycoplasma* spp.

UTIs: urinary tract infections.

## Data Availability

No new data were created or analyzed in this study. Data sharing is not applicable to this article.
